# Antitumor effects of dauricine on sorafenib-treated human lung cancer cell lines via modulation of HIF-1α signaling pathways

**DOI:** 10.1007/s12032-025-02679-4

**Published:** 2025-04-10

**Authors:** Eman K. Teleb, Radwa A. Mehanna, Nagwa M. Assem, Maha E. Houssen

**Affiliations:** 1 Biochemistry Department, Faculty of Pharmacy, Damanhour University, Damanhour, 22511 Egypt; 2 Medical Physiology, Faculty of Medicine, Alexanderia University, Alexanderia, Egypt; 3 Center of Excellence for Research in Regenerative Medicine and Its Applications, Faculty of Medicine, Alexandria University, Alexanderia, 22511 Egypt; 4 Department of Biochemistry, Medical Research Institute, Alexandria University, Alexanderia, 22511 Egypt

**Keywords:** Non-small cell lung cancer, Hypoxia-inducible factor, Sorafenib, Dauricine

## Abstract

The majority of lung cancer cases are non-small cell lung cancer (NSCLC) which continues to be a serious global health concern. Hypoxia-inducible factor (HIF-α) pathway is a promising therapeutic target because it has a vital function in advanced non-small cell lung carcinoma. Antiangiogenic multi-kinase inhibitor, sorafenib may have a part in regulating HIF signaling in cancer. As a result, there is now more interest in employing it to target hypoxia-driven pathways in non-small cell lung cancer, especially when paired with natural bioactive products such as dauricine which is a naturally occurring alkaloid molecule targets multiple cellular pathways to provide strong anticancer effects. To examine molecular impacts of combining dauricine with sorafenib on HIF-mediated signaling pathways in human lung cancer cell lines. Cell viability was assessed using MTT assay in A549 and H1975 lung tumor cell lines. Levels of key proteins (AKT, mTORC1, HIF-1 α, ERK, VEGF, Cyclin-D1, BCL2, and E-Cadherin) were measured by ELISA.A colorimetric test was utilized to assess the activity of caspase-3, as a marker of apoptosis. qRT-PCR was employed to identify PI3K and VEGFR2 genes expression. Combination of sorafenib and dauricine significantly enhanced cytotoxicity compared to either agent alone. This combination also led to a marked reduction in VEGFR2, PI3K expression and VEGF, AKT, mTOR, HIF-1α, BCL2, ERK and E-Cadherin, and Cyclin-D1 levels. In addition, there was a significant increase in caspase-3 activity. Dauricine potentiates antitumor effects of sorafenib in human NSCLC by modulating HIF-1α-mediated pathways that are involved in several cancer hallmarks. This combination shows promise as a potential lung cancer treatment approach.

## Introduction

Worldwide, lung cancer is the primary reason for mortality associated with cancer. Non-small cell lung carcinoma (NSCLC) is the most prevalent kind among its different variants [1, 2], and it is frequently detected at an advanced stage, which restricts treatment options and prognosis [3]. Lung cancer is complicated by its heterogeneity and propensity to become resistant to conventional treatments [4, 5].

Targeted treatment has improved the management of advanced non-small cell lung carcinoma in the last few years by focusing on particular molecular pathways necessary for tumor development and survival [6]. One of such mechanisms which controls cellular reactions to low oxygen levels, a prevalent situation in the tumor microenvironment [7] is HIF-1α (hypoxia-inducible factor 1α) oncogenic pathway [8]. In addition, besides encouraging tumor aggressiveness and metastasis, hypoxia is another important factor in non-small cell lung tumor chemotherapy resistance [9].

A transcription factor complex made up of HIF-1α and HIF-1β subunits controls the HIF-1α pathway, which mediates angiogenesis, apoptosis, evasion, proliferation, and the epithelial-mesenchymal transition [10]. Genes involved in cell growth, formation of new blood vessels(angiogenesis), apoptosis, and chemotherapy resistance become transcriptionally active when hypoxia-inducible factor 1α stabilizes and forms heterodimer with HIF-1β under hypoxic conditions [11, 12]. This adaptive response promotes tumor growth and treatment evasion by enabling cancer cells to endure oxygen-deficient conditions [13, 14].

Sorafenib originally recognized for its antiangiogenic effects on Raf kinases and receptor tyrosine kinases [15]. Sorafenib has demonstrated promise in modifying the HIF pathway, according to studies, sorafenib can impair hypoxia-driven signaling cascades by lowering HIF-1α expression and activity [16].

A major clinical problem that frequently results in therapy failure is sorafenib resistance [17]. To combat this resistance, researchers have been investigating a number of approaches, such as using natural products [18]. A possible strategy for increasing sorafenib's effectiveness and reducing its resistance is the use of natural compounds made from plants, animals, or microbes, these substances can be utilized as adjuvant therapy to target various resistance pathways, which may improve clinical results [19, 20].

Dauricine, a natural alkaloid, found in many plant species, particularly those in the Menispermaceae family [21]. Its anti-inflammatory, antiarrhythmic, and protective effects against cerebral damage have led to its traditional use [21, 22]. Numerous studies have shown its strong anticancer properties, such as inducing apoptosis, antiproliferation, and anti-angiogenesis, even though its involvement in cancer treatment and prevention is still a developing field of study [23].

In light of these findings, the purpose of this work was to examine the molecular effects of dauricine on HIF-mediated cancer signaling pathways in sorafenib-treated human lung carcinoma cell lines.

## Material and methods

### Chemicals

Sorafenib tosylate (≥ 96% HPLC) (Cat. No. #SML2653) acquired from Sigma Aldrich in St. Louis, MO, USA. In order to prepare the required concentration (5 mM), it was dissolved in 2.1514 mL dimethyl sulfoxide (DMSO). To prepare final concentration (5 mM) from Dauricine (Cat. No. #80,419) (Sigma Aldrich), it was dissolved in 0.3201 mL DMSO which then kept stored at − 20 ℃. Fetal Bovine Serum (FBS) (Cat. No. #S1300) purchased from BiowestCo (Nuaillé, France). SERVA Electrophoresis GmbH (Heidelberg, Germany) supplied 3-(4,5-dimethylthiazolyl-2)-2,5-diphenyltetrazolium bromide (MTT) and Dimethyl sulfoxide (DMSO). Trypsin, Dulbecco's modified Eagle’s medium (DMEM) High glucose (Cat. No. L0103) and Dulbecco’s phosphate-buffered saline (PBS) (Cat.No.#L0615) were acquired from Biowest.st. Penicillin and streptomycin antibiotic mixtures were purchased from El Maadi Medical Supplies (Cairo, Egypt). Pharmaceutical Chemicals Co. in Cairo, Egypt was the source of ethanol (70%).

### Cell lines and cell culture

A549 and H1975 two distinct two humans non -small lung cancer (NSCLC) cell lines were acquired from the American Type Culture Collection (ATCC, USA). The Cells were cultured in DMEM high glucose enhanced with 1% penicillin/streptomycin and 10% FBS. After that cells incubated at 37 ℃ in a humidified 5% (*v*/v) CO_2_ environment with 5% (*v*/v) CO_2_. Media were replaced regularly after three to four days, when cells achieved 80–90% confluence cells were passaged. The cells were cultured in 75 cm two flasks (five passages after thawing) [24].

### Assessment of cell viability (MTT Assay)

The proliferation effects of sorafenib, dauricine, and their combination on human NSCLC A549 and H1975 cell lines were determined through MTT assay. After seeding 104 cells per well in 96-well plates, the cells were incubated at 37 °C overnight. 100 μl of media containing sorafenib at 0, 1, 2.5, 5, 10, 20, 40 μM and dauricine 0, 1, 2.5, 5, 10, 20, 40 μM were added after the old media aspiration and assayed in triplicate for both cell lines. Medium was removed from the plate by aspiration following cells incubation for 48 h at 37 ℃. Then twenty microliters of MTT solution (5 mg/mL in PBS) were added to the plate and incubated for four hours. To dissolve purple formazan, 120 µl of dimethyl sulfoxide (DMSO) was added after removal of supernatant. Enzyme-linked immunosorbent assay device (Bio-Tek, Inc., Winooskie, VT, USA) was utilized to read absorbance at wavelength 570 nm, with the absorbance at 630 nm as the background correction) [24]. The percentage of untreated controls was used to represent Cell viability. Every experiment was run at least three trials.

### Experimental cell design or cell lines treatment protocol

Both cell types were seeded individually in 15 T-75 flasks and incubated overnight. In the following day, cells were separated into 4 distinct groups and treated as following: first group left untreated (control), and the second group treated with sorafenib (8.8 μM for A549 and 8.3 μM for H1975), and the third group treated with dauricine (15.72 μM for A549 and 17.4 μM for H1975). We used a dose equal to its half IC50 for two drugs on each cell type in the second and third groups. Finally, the fourth group received a combination treatment of sorafenib and dauricine at a dose equal to its quarter IC50 for A549 cell (4.4 μM sorafenib + 7.86 μM dauricine) and half IC50 for H1975 cell (8.3 μM sorafenib + 17.4 μM dauricine). Cells from four groups were collected after 48 h of incubation and kept as a separated aliquot. The aliquots were stored at a frigid – 80 °C until they were used in the next investigation.

### Cell lysates preparation

Boster Biological Technology (Pleasanton, CA, USA) provided the RIPA lysis buffer (AR0105) which was used to create cell lysates. As directed by the manufacturer, cell pellets were mixed with 0.5 mL of RIPA buffer, then vortexed briefly. After 30 min of ice incubation, these pellets were centrifuged at 14,000 × g for 10 min in order to eliminate cell debris. The supernatants were transferred to a new tube and kept at − 20 ℃ for additional analysis.

### Biochemical analysis

#### Biomarker analysis via sandwich ELISA technique

In A549 and H1975 cells, sandwich ELISA technique was used following the manufacturer’s instructions to quantify multiple tumor biomarkers including Cyclin-D1 (Catalog #HUFI00736, My BioSource, CA, USA), mTORC1 (Catalog # MBS1600031MyBioSource, Thermo science, USA), Hypoxia-Inducible Factor 1 Alpha (HIF-1α Catalog #E-EL-H6066), human Vascular Endothelial Growth Factor (VEGF) (Catalog #DVE00, My BioSource, USA R&D Systems, Bio-Techne Ltd), p-AKT (Catalog # KHO0111, Thermo science), E-Cadherin(Catalog #E-EL-H0014, Biosource Elabscience, USA), pERK1/2(My BioSource Catalog # E-EL-H1698, Elabscience, USA), and Bcl2(My BioSource Catalog #E-EL-H0114, Elabscience, USA).

#### Colorimetric assay of caspase -3 activity

In accordance with the manufacturer's instructions, active caspase-3 was determined using the Colorimetric kit (Product Code CASP-3-C, Sigma Aldrich, USA) by releasing the p-nitroaniline (pNA) moiety as a result of the hydrolysis of the peptide substrate acetyl-Asp-Glu-ValAsp p-nitroanilide (Ac-DEVD-pNA) by caspase-3. The absorption of p-nitroaniline is high at 405 nm (εmM = 10.5). The calibration curve made using specific pNA solutions is used to determine the concentration of the pNA emitted from the substrate.

#### Quantitative real-time polymerase chain reaction (qRT-PCR)

As directed by the manufacturer, the quantitative real-time reverse transcription polymerase chain reaction (qRT-PCR) technique was used. First, TRIzol™ Plus RNA Purification Kit (Cat. No. 12183555, Thermo Fisher Scientific) was employed to separate RNA from cell lines. After that High-Capacity cDNA Reverse Transcription Kit, 200 reactions (Cat. No.4368814, Thermo Fisher Scientific, USA) were utilized to reversed-transcribed the RNA using equivalent concentrations of isolated RNA. Finally, qRT-PCR system was used to measure gene expression levels for two genes VEGFR2 and PI3K by using Maxima SYBR Green qPCR Master Mix (2x) kit (Cat. No: K0252, Thermo Fisher Scientific) using the following primer pairs sequence (Table 1).

**Table 1:** Sequence of qRT-PCR primer pairs

Gene	Forward primer sequence	Reverse primer sequence
VEGFR2	5'CCTTGGAGCATCTCATCTGT3'	5'GTGGATACACTTTCGCGATG3'
PI3K	5'AGGTGATCGAGAGAGGCA3'	5'AGGTGATCGAGAGAGGCA3'
ACTB (β-actin reference gene)	5'CACCATGGATGATGATATCGC 3'	5'GAATCCTTCTGACCCATGCC3'

ACTB (reference gene) was used as a housekeeping gene to determine the changes in VEGFR2 and PI3K gene expression. Three evaluations were performed on every sample

### Statistical analysis

The findings were presented as the mean ± standard deviation (SD), and each of the experiments was conducted three times per group. GraphPad Prism 8.0 software (GraphPad Software, USA) was used to analyses several differences among both control and treatment groups, using one-way ANOVA test followed by post* hoc* Tukey’s technique. At, a *P* value > 0.05. The outcomes became statistically significant.

## Results

### Cytotoxic effect of sorafenib and dauricine on NSCLC (A549 and H1975) cell lines

**Fig. 1:**
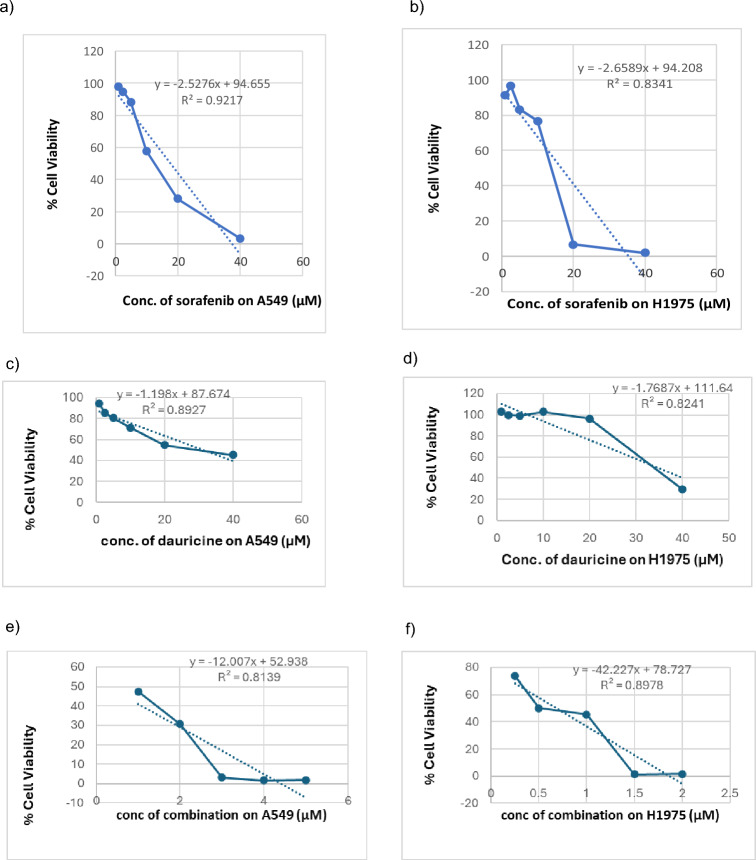
The effect of sorafenib (1–40 μM), dauricine (1–40 μM), and sorafenib–dauricine combination on the viability of A549 and H1975 NSCLC

Sorafenib showed cytotoxic effect on both A549 and H1975 cells, with an IC50 of 17.6 μM for A549 and 16.6 μM for H1975. While dauricine showed cytotoxic effect with IC50 31.44 μM and 34.8 μM for both A549 and H1975, respectively, in treated cells. Furthermore, sorafenib and dauricine combination at the same concentration ranges showed cytotoxicity with a combined IC50 (4.4 μM for sorafenib + 7.86 μM for dauricine) for A549 cells, and (8.3 μM for sorafenib + 17.4 μM for dauricine) for H1975 cells (Fig. 1).

### Effect of sorafenib, dauricine and their combination on PI3K expressions and p-AKT levels

**Fig. 2:**
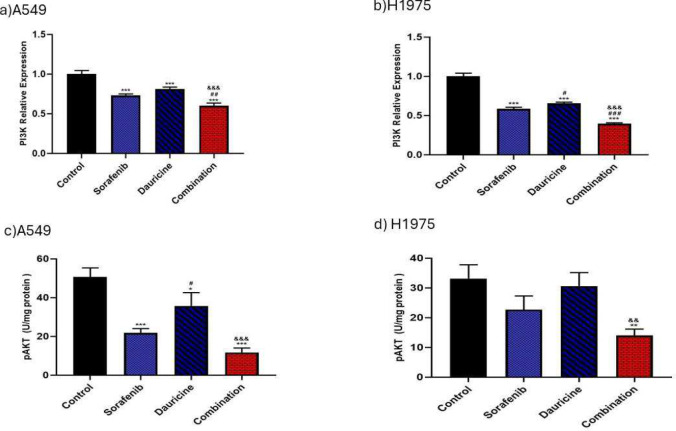
**a** and **b** Effects of sorafenib, dauricine, and combination on PI3K expression in A549 and H1975 cells. **c** and **d** Effects of sorafenib, dauricine, and combination on p-AKT level in A549 and H1975 cells. Statistically significant differences between groups were designated as *p < 0.05 vs. control, #vs. sorafenib, & *p* < 0.05 vs. dauricine

A notable reduction in PI3K l gene expression levels in sorafenib, dauricine, and combination groups were detected in comparison to control group for both cell lines (*p* < 0.05). A significant reduction in P-AKT levels was detected in sorafenib, dauricine, and combination groups compared to control group in A549 cell line (p < 0.05). Combination groups demonstrated a significant reduction in PI3K expression compared to either sorafenib or dauricine alone, in both cell lines (*p* < 0.05). Combination group had a significant decrease in p-AKT levels compared to either control or dauricine alone (*p* < 0.05). **(**Fig. 2).

### Impact of sorafenib, dauricine, and their combination on mTORC1and HIF-1α levels

**Fig. 3:**
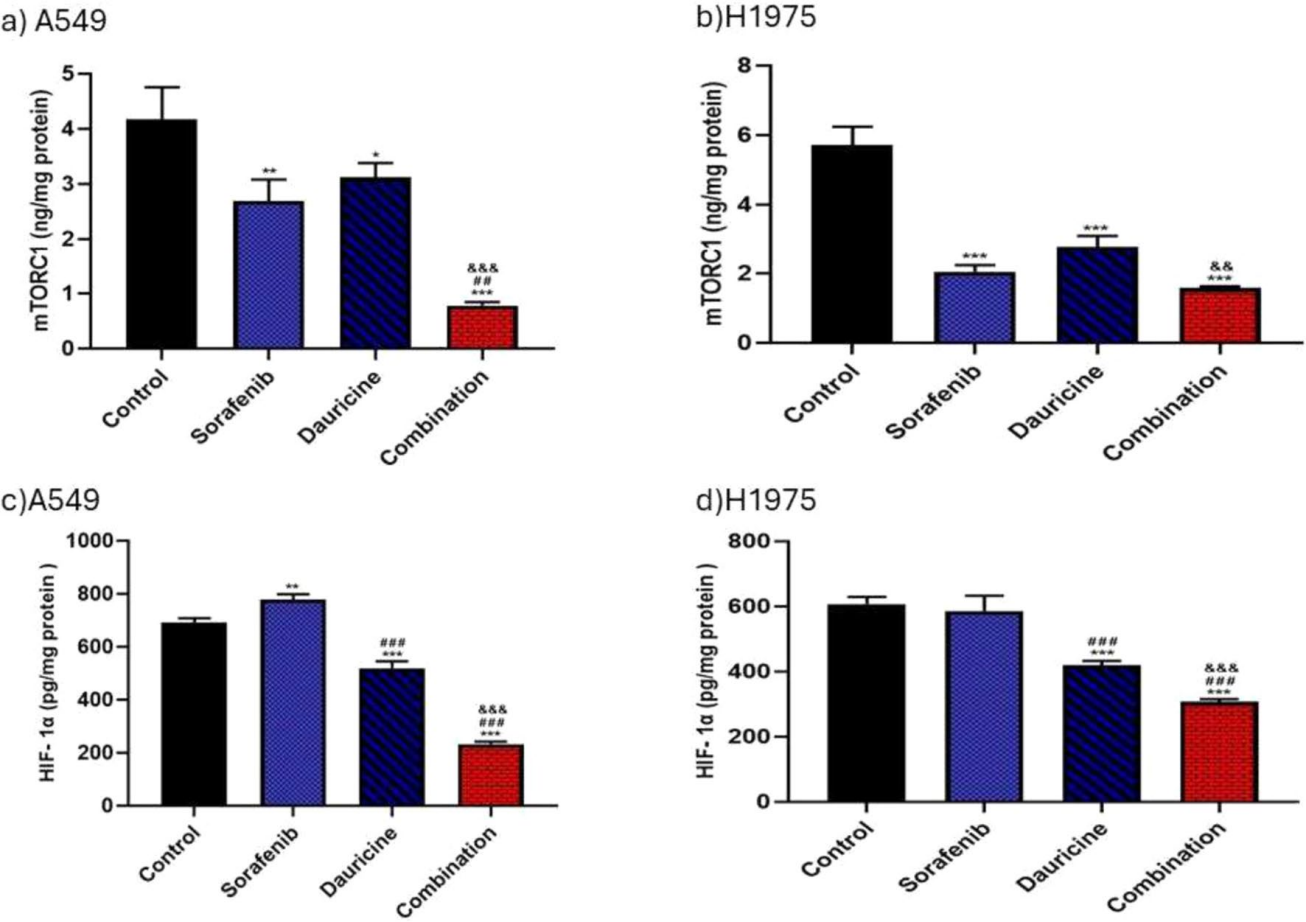
**a** and **b** Effects of sorafenib, dauricine, and combination on mTORC1 level in A549 and H1975 cells. **c** and **d** Effects of sorafenib, dauricine, and combination on HIF-1α1 in A549 and H1975 cells. Statistically significant differences between groups were designated as *p < 0.05 vs. control, #vs. sorafenib, & *p* < 0.05 vs. dauricine

The combination group’s mTORC1 levels were much lower when compared to dauricine and untreated groups in both cell lines (*p* < 0.05). Combination groups indicated a significant inhibition of HIF-1α levels compared to sorafenib, dauricine, and control groups in both cell lines (*p* < 0.05).** (**Fig. 3).

### Effect of sorafenib, dauricine, and the combination of them on p-ERK

**Fig. 4:**
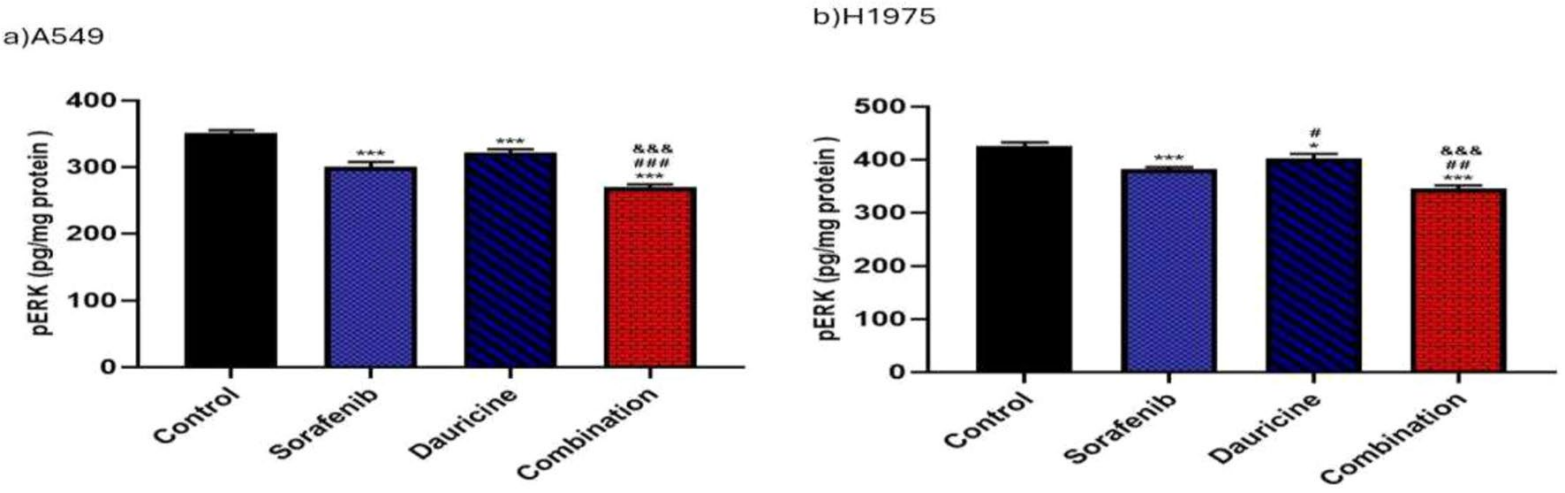
**a** and **b** Effects of sorafenib, dauricine and combination on p-ERK level in A549 and H1975 cells. Statistically significant differences between groups were designated as *p < 0.05 vs. control, #vs. sorafenib, & *p* < 0.05 vs. dauricine

Sorafenib, dauricine, and combination groups showed an inhibitory effect on p-ERK levels compared with control group in both cell lines (*p* < 0.05). Combination group revealed a significant reduction in p-ERK levels compared with sorafenib or dauricine in both cell lines (*p* < 0.05). (Fig. 4).

### Effect of sorafenib, dauricine, and their combination on Cyclin-D1 as a proliferation marker

**Fig. 5:**
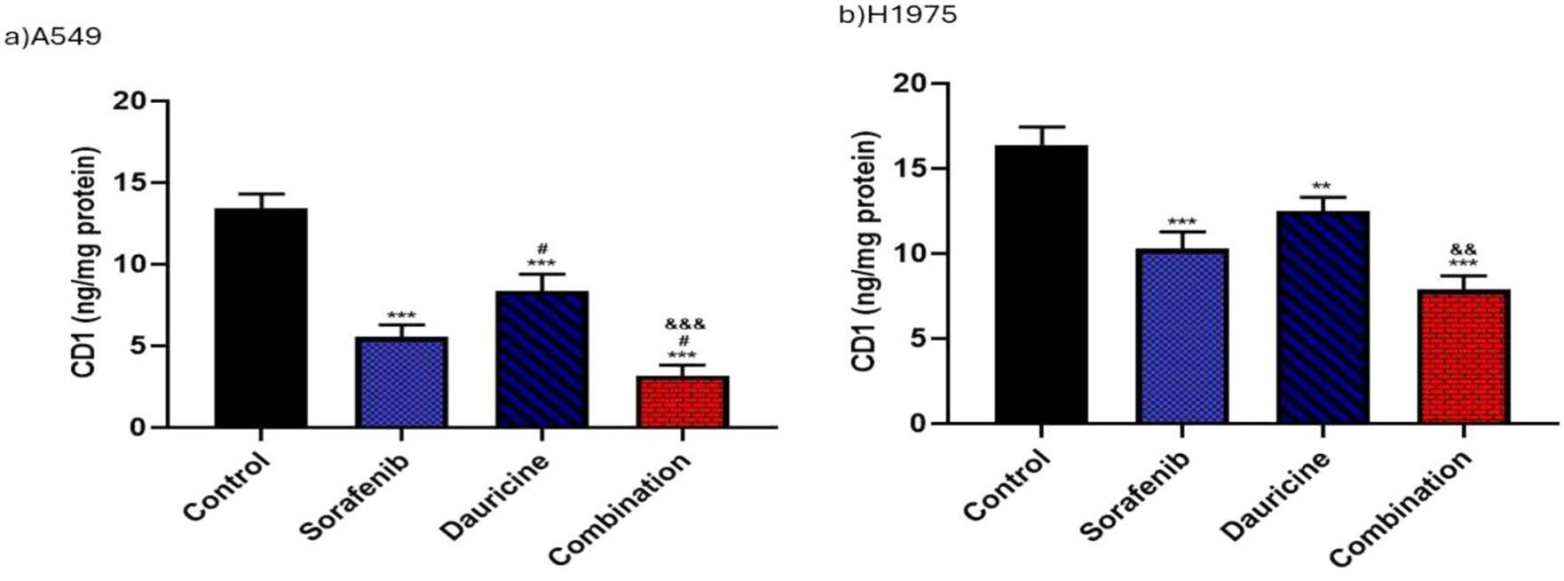
**a** and **b** Effects of sorafenib, dauricine, and combination on Cyclin-D1 in A549 and H1975 cells. Statistically significant differences between groups were designated as *p < 0.05 vs. control, #vs. sorafenib, & *p* < 0.05 vs. dauricine

Sorafenib, dauricine, and their combination group revealed a remarkable decline in cyclin-D1 levels when compared to control group in both cell lines (*p* < 0.05). Cyclin-D1 levels in A549 cells were significantly lower in the combination group than in the sorafenib or daucine group alone but in H1975 cells, the combination group's cyclin-D1 levels were significantly lower than those of the dauricine group alone (*p* < 0.05). (Fig. 5).

### Impact of sorafenib, dauricine, and their combination on VEGF level\VEGFR2 expression as angiogenesis marker

**Fig. 6:**
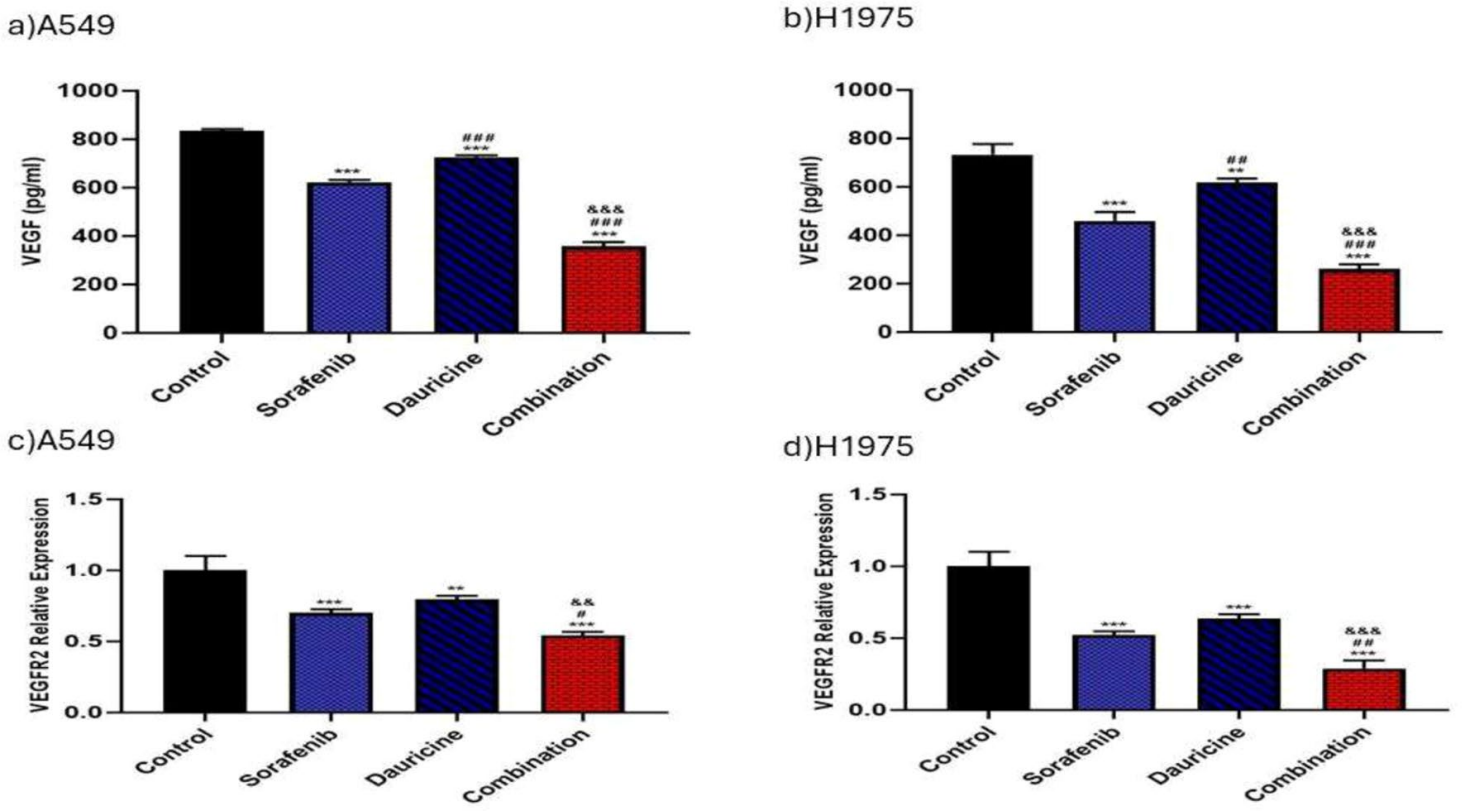
**a** and **b** Effects of sorafenib, dauricine, and combination on VEGF levels in A549 and H1975 cells. **c** and **d** Effects of sorafenib, dauricine, and combination on VEGFR2 gene expression in A549 and H1975 cells. Statistically significant differences between groups were designated as *p < 0.05 vs. control, #vs. sorafenib, & *p* < 0.05 vs. dauricine

Sorafenib, dauricine, and combination groups revealed a significant decrease in VEGF levels and VEGFR2 gene expressions compared with control group in both cell lines (*p* < 0.05). Combination group had a significant decrease in VEGF levels and VEGFR2 gene expressions when comparing with sorafenib and dauricine groups alone (*p* < 0.05). (Fig. 6).

### Impact of sorafenib, dauricine and their combination on BCL2 levels

**Fig. 7:**
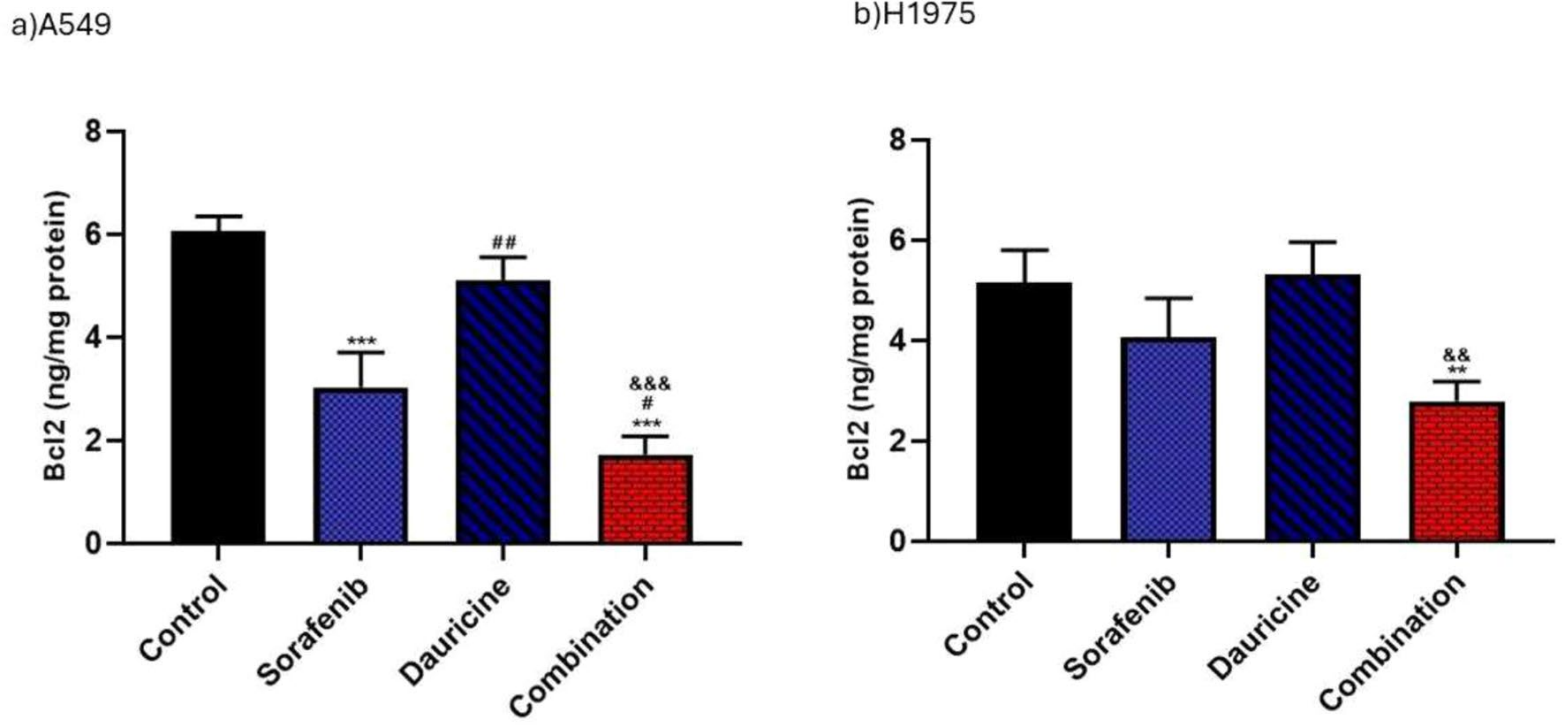
**a** and **b** Effects of sorafenib, dauricine, and combination on BCL2 in A549 and H1975 cells. Statistically significant differences between groups were designated as *p < 0.05 vs. control, #vs. sorafenib, & *p* < 0.05 vs. dauricine

Combination group significantly reduces BCL2 levels in comparison to dauricine, sorafenib, and untreated groups in A549 cell (*p* < 0.05). In H1975 cell line, combination group's BCL2 levels were significantly lower than those of untreated \or dauricine groups (*p* < 0.05). (Fig. 7).

### Effect of sorafenib, dauricine and their combination on caspase-3 activities

**Fig. 8:**
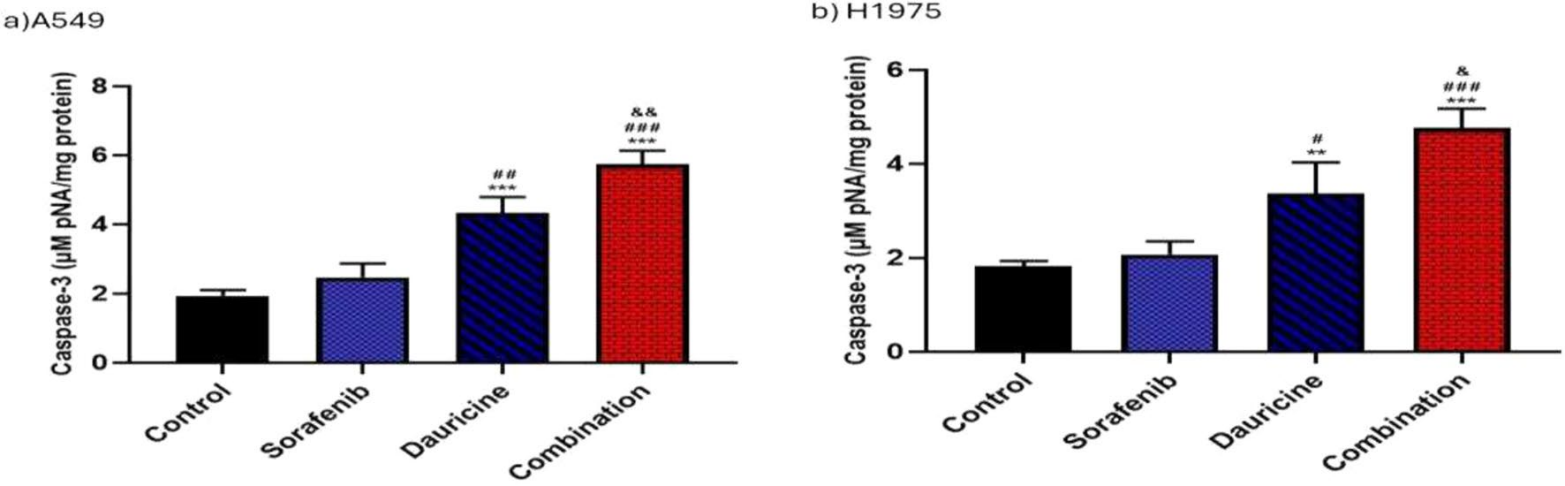
**a** and **b** Effects of sorafenib, dauricine, and combination on caspase-3 activity in A549 and H1975 cells. Statistically significant differences between groups were designated as *p < 0.05 vs. control, #vs. sorafenib, & *p* < 0.05 vs. dauricine

The combination group significantly elevated caspase-3 activity relative to the sorafenib, dauricine, and untreated groups in both cell lines (*p* < 0.05). (Fig. 8).

### Effect of sorafenib, dauricine, and their combination on epithelial–mesenchymal transition (EMT) marker E-Cadherin

**Fig. 9:**
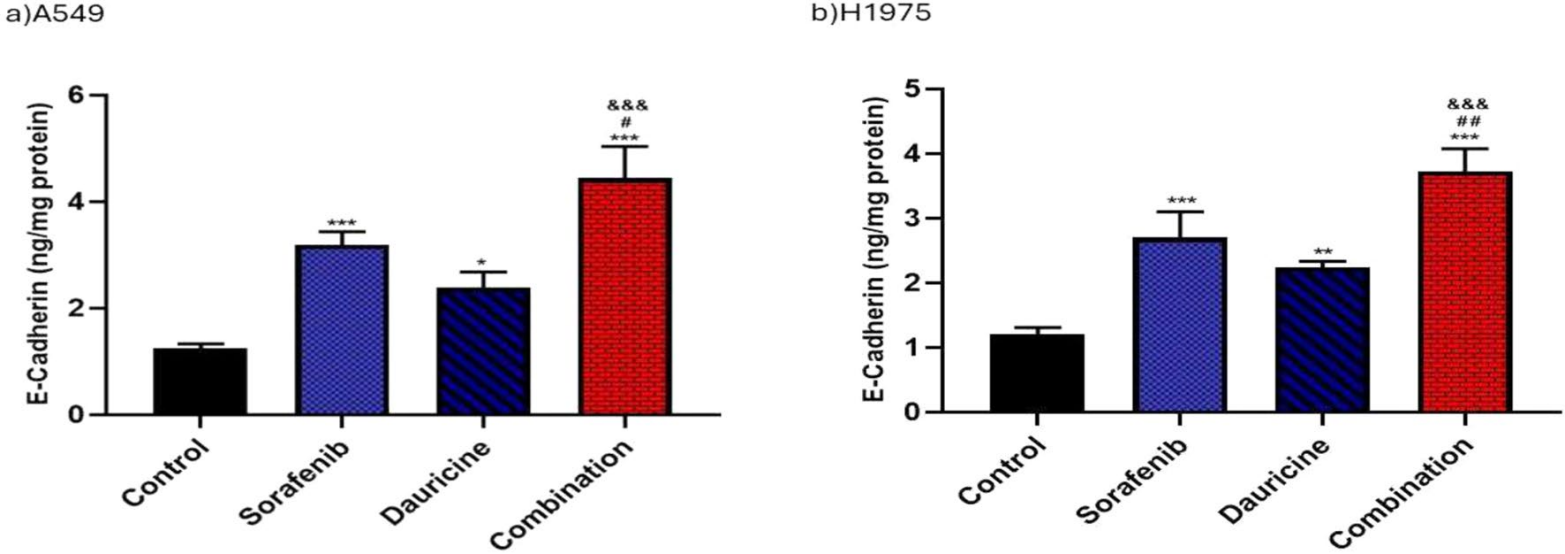
**a** and **b** Effects of sorafenib, dauricine, and combination on E-cadherin level in A549 and H1975 cells. Statistically significant differences between groups were designated as *p < 0.05 vs. control, vs. sorafenib, & *p* < 0.05 vs. dauricine

Sorafenib, dauricine, and their combination groups exhibited a significant elevation in E-cadherin levels as compared to untreated group in both cell lines (*p* < 0.05). E-cadherin values in the combination groups were significantly higher than those in the sorafenib/dauricine groups individually (*p* < 0.05). (Fig. 9).

## Discussion

Globally, lung cancer continues to be the primary cause of cancer mortality [25]. It is the cancer that is most frequently recognized after breast malignancy, making up about 11.4% of all new cancer cases each year [25]. Lung cancer is frequently diagnosed at advanced stages, when treatment choices are restricted, which contributes to this high fatality rate [3].

An essential element in initiation of non-small cell lung carcinoma (NSCLC) is hypoxia-inducible factor 1α (HIF-1α), which is a transcription variable reacts to oxygen deprivation, or low oxygen levels in the cellular environment [26], a common condition in solid tumors like lung cancer [27]. The hypoxic conditions inside tumors often cause HIF-1α to be increased in lung cancer [28]. Numerous genes that support tumor survival, angiogenesis, metastasis, and invasion are activated as a result of this overexpression [29].

In light of these results, the purpose of this study was to examine the molecular mechanisms and anticancer impacts of dauricine on H1975 and A549, two human lung tumor cell lines treated with sorafenib. Specifically, the study focused on the modulatory effects of this combination on the HIF-1α signaling pathways, which mediate angiogenesis, apoptosis, evasion, proliferation, and the transition from epithelial to mesenchymal stage (EMT) in cancer cell lines. As far as we are aware, this is the first study to look into how the combination of dauricine and sorafenib affects human NSCLC cell lines when used as chemotherapy.

According to the results of the MTT experiment, sorafenib and dauricine together had a greater cytotoxic effect at lower dosages than either drug alone. This result is in line with past research showing that the antiproliferative effect of combined therapy may be partly related to growth-inhibitory effects of sorafenib on NSCLC cells [30] and the possible cytotoxic effect of dauricine through apoptosis induction, cell proliferation inhibition, and metastasis [31, 32].

The present study revealed that HIF-1α exhibited high levels in the control groups of both cell lines. This elevated expression and activation of HIF-1α in cancerous cells is attributable to the dysregulation in central signal transduction axes, such as PI3K-AKT-mTOR axis, which is actively operating in tumor cells and triggers downstream signaling leading to an increase in HIF-1α transcription factor translation [33, 34]. In addition, HIF-1α was also regulated by the overactivated oncogenic ERK cascade (extracellular signal-regulated kinase) in the untreated control groups [35]. However, in both cell lines, the combination groups revealed a notable decline in HIF-1α levels in contrast to either control, dauricine, or sorafenib groups. This decrease can be explained by the combination's possible inhibitory effects on the ERK and PI3K/AKT/MTOR pathways [32, 36].

ERK1 and ERK2 are extracellular Signal Regulated Kinases are phosphorylated, activated, and translocated into the nucleus in response to hypoxia [37, 38]. In contrast to control groups and the sorafenib or dauricine groups alone, we showed in this study that the sorafenib and dauricine combination groups significantly inhibited p-ERK levels in two cell lines H1975 and A549. This result aligns with prior investigations shown that sorafenib inhibited Raf/MEK/ERK signaling in human neuroblastoma cell lines and HCC, hence, suppressing the phosphorylation of ERK signaling [16, 39]. Furthermore, in colon cancer, dauricine had little effect on ERK1/2 phosphorylation [31].

With respect to cyclin-D1, a proliferation marker, the current investigation concluded that dauricine and sorafenib together suppressed cell growth in both A549 and H1975 cell lines, as reflected by a decrease in levels of cyclin-D1 relative to untreated groups. cyclin-D1 elevation in control groups can be attributed to HIF-1α which activated the AKT/Cyclin-D1 pathway stimulate the tumor development in osteosarcoma cells [40]. Conversely, the inhibitory effect of the combination group may be attributed to the effects of dauricine and sorafenib on cyclin-D1. Dauricine-induced cell cycle arrest at the G0/G1phase was associated with the reduction of cyclin-D1 expression in renal tumor cells and colon cancerous cells [31, 32]. In addition, Sorafenib had an effective antiproliferative impact by reducing cyclin-D1 expression, leading to cell cycle arrest in combination with other drugs in lung cancer [41].

In reference to VEGF, an angiogenic marker, this research demonstrated that combo of sorafenib–dauricine had a strong antiangiogenic influence in both A549 and H1975 cell lines, as evidenced by a decrease in PI3K, VEGFR2 gene expression, and a decrease in AKT, mTOR, HIF-1α, and VEGF levels. These findings were attributed to sorafenib's antiangiogenic effect, which derives from the inhibition of the HIF-1α/VEGF pathway [16, 42, 43]. Furthermore, studies conducted in vitro have demonstrated that sorafenib is a strong inhibitor of pro-angiogenic VEGFRs 2/3 [44]. In addition, by encouraging the breakdown of hypoxia-inducible factor 1α(HIF-1α) protein and preventing the stimulation of the PI-3 K/Akt/mTOR/HIF-1α/VEGF axis, dauricine reduces the creation of HIF-1α protein, which in turn prevents angiogenesis in human breast cancer cell lines [45].

Concerning apoptosis markers, it is well established that hypoxia and apoptosis are closely related processes, as Bcl2 and HIF-1α can mutually regulate each other's activity [46, 47]. To promote cell survival in hypoxic environments, HIF-1 promotes the production of many antiapoptotic BCL2 proteins [48]. Furthermore, Bcl2 can prevent cell death by stabilizing the hypoxia-inducible factor-1α protein, which increases HIF-1 activation process [49]. Gene expression of pro\antiapoptotic Bcl2 proteins is controlled by HIF-1α [50]. In contrast to the control group and cells treated with either sorafenib or dauricine alone, this study indicated that NSCLC cells injected with sorafenib and dauricine combo exhibited a marked drop in Bcl2 values, a protein that inhibits apoptosis. This result is in agreement with past research, which found a strong positive relationship between HIF-1 and Bcl2 expression in neuroblastoma [51].

Comparing NSCLC cells treated with sorafenib plus dauricine to cells treated with either sorafenib or dauricine alone, this study found a substantial increase in caspase-3 activity, an apoptotic enzyme. This impact could be partially ascribed to the intrinsic mechanism-induced apoptotic action of dauricine in RCC cells, which is linked to a downregulation in gene expression in Bcl2 antiapoptotic protein and the induction of caspase-9 and caspase-3 [30].

With respect to EMT, both NSCLC cells lines that received sorafenib along with dauricine exhibited considerable rise in E-cadherin, a hallmark of the epithelial-mesenchymal transition (EMT), in comparison to control groups and sorafenib or dauricine groups alone. This effect is partially attributed to the reduction of PI3K-Akt and HIF-1α expression which led to EMT inhibition [52]. Furthermore, prior research has shown that sorafenib and its combination with other drugs inhibit EMT in A549 cells. This inhibition is reflected with an increase in mRNA and protein expression of E-cadherin, which inhibits the metastatic ability of NSCLC cells [53].

## Conclusion

In conclusion, the present study demonstrates that dauricine significantly enhances the antitumor effects of sorafenib in NSCLC known as non-small cell lung cancer by modulating key cancer signaling pathways, particularly those mediated by HIF-1α**.** The combination of sorafenib and dauricine effectively inhibits the HIF-1α**,** VEGF**,** PI3K/AKT/mTOR, and ERK pathways, reduces levels of BCL2, a protein that prevents apoptosis, and decreases Cyclin-D1 expression, which is associated with cell proliferation. Moreover, the combination therapy increases caspase-3 activity, promoting apoptosis. These findings suggest that targeting hypoxia-driven pathways via the combined use of sorafenib and dauricine could be a viable therapeutic approach for progressed NSCLC, potentially combating treatment resistance and improving patient outcomes. Further preclinical and clinical studies are recommended to validate the efficacy and safety of the sorafenib-dauricine combination in NSCLC**.** Mechanistic studies should clarify their interaction with the HIF-1α pathway, and exploration of this combination with other therapies may reveal synergistic effects. In addition, optimizing drug delivery and exploring this combination in other hypoxia-related cancers could expand its therapeutic potential.

## Data Availability

The data that support the findings of the current study are available from corresponding author upon request.
